# Surgical debridement and split-thickness skin grafting in debilitating bilateral lower extremity calcinosis cutis: A case report

**DOI:** 10.1016/j.jdcr.2026.03.004

**Published:** 2026-03-13

**Authors:** Eli Luna, Jenna Thomason, Jeffrey Friedrich, Andrea Kalus

**Affiliations:** a Department of Medicine, University of Washington, Seattle, Washington; b Division of Rheumatology, University of Washington, Seattle, Washington; c Division of Plastic Surgery, University of Washington, Seattle, Washington; d Department of Dermatology, University of Washington, Seattle, Washington

**Keywords:** calcinosis cutis, intravenous immunoglobulin, scleroderma, split-thickness skin graft, surgery

## Introduction

Calcinosis cutis (CC) results when insoluble calcium salts deposit in cutaneous tissues.^1^ The dystrophic subtype is the most common and stems from local tissue injury or extra-cellular matrix protein defects.^2^ Dystrophic calcification is strongly associated with autoimmune diseases such as systemic sclerosis, particularly limited cutaneous systemic sclerosis (lcSSc), and dermatomyositis.^3^ Therapeutic options for calcinosis cutis remain limited, and response to pharmacologic interventions is variable.^1^^,^^3^ Surgical management has traditionally been reserved for small, localized lesions due to concerns regarding wound healing, recurrence, and functional compromise. Surgery is rarely considered for patients with extensive or circumferential disease. Ulceration overlying calcinosis cutis is especially difficult to manage because the areas of ulceration are very unlikely to heal without debriding the calcinosis completely. We present a novel report of successful bilateral lower extremity circumferential debridement and split-thickness skin grafting (STSG) in a patient with CC and overlying ulcers who exhausted multiple pharmaceutical treatment interventions. This contribution to the literature highlights the effectiveness of surgical management when other treatments fail and emphasizes that complete debridement may be necessary to facilitate wound healing.

## Case study

The patient is a 54-year-old woman with lcSSc and bilateral circumferential calcifications of her lower legs and progressive ulceration. Medical records revealed a 10-year history of lcSSc with positive antinuclear and anti-centromere antibodies. Other clinical features included telangiectasias, gastroesophageal dysmotility, and Raynaud's phenomenon. The initial ulcer appeared midway down the left lower lateral leg measuring 1 to 2 cm and revealed underlying visible trabecular-like calcinosis. Months of wound care management included topical collagenase with compression wraps, but after no improvement, her rheumatologist referred her to a plastic surgeon who debrided the calcinosis in a 3 cm × 3 cm area on her left leg with split thickness skin grafting to cover. She was then referred to our combined dermatology rheumatology clinic.

On our initial exam, the central skin grafted area was well adhered with no calcinosis but there was peripheral progressive ulceration in a ring like configuration around the healed skin graft. The area now measured 8 cm × 6.5 cm with visible calcinosis in the peripheral areas of the ulcer. Meticulous wound care was continued along with sodium thiosulfate (STS) therapy, first with intralesional STS, then compounded STS in zinc paste, STS soaked wound compresses, and eventually 6 months of intravenous STS therapy. During this time there was no improvement in the calcinosis and new ulcers appeared now involving both lower legs. Minocycline, colchicine, and bisphosphonates were trialed sequentially and discontinued due to lack of clinical benefit.

Medical treatment for lcSSc included mycophenolate and a trial of hydroxychloroquine. Her Raynaud’s phenomenon was managed with multiple agents including nifedipine and botulinum toxin injections. Sildenafil, later transitioned to tadalafil, was used for pulmonary hypertension with additional peripheral benefit. She also received pentoxifylline and dipyridamole for peripheral vasculopathy.

**Fig 1:**
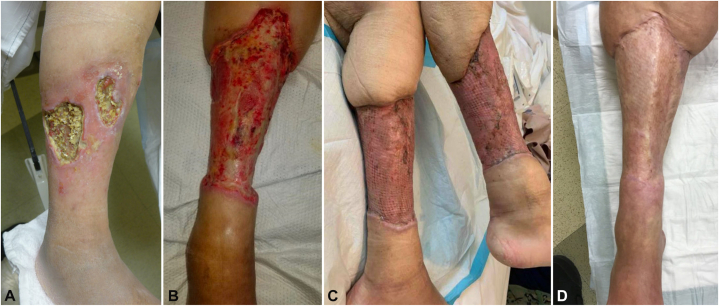
Patient with limited scleroderma and circumferential calcinosis cutis of both lower legs. **A,** Shows two ulcers with widespread calcinosis in the ulcer bed (right leg). **B,** Demonstrates the excisional debridement to muscle of the right leg. **C,** Demonstrates an immediate postoperative bilateral clinical image of the grafted sites, and **D,** shows the healed result (right leg) 17 months after surgery.

Despite these interventions over almost 2 years, the progressive ulcers, pain, and high burden of care required for wound care management contributed to the patient’s deteriorating quality of life. To continue working and to sustain her desired active lifestyle, the patient expressed interest in bilateral amputation. After evaluation by plastic surgery, 2 approaches were considered: bilateral below-knee amputation versus circumferential soft tissue reconstruction. The plastic surgery team proceeded with circumferential soft tissue reconstruction leaving adequate tissue for amputation if necessary. The patient underwent a two-stage procedure. (1) Complete and circumferential excision of calcinosis from both legs with placement of Porcine xenograft. (2) After 1 week, xenograft removal and autologous split thickness skin grafting from bilateral thighs (Fig 1, *B* and *C*).

Postoperatively, the grafts healed well. At the distal aspects, just above the ankle, serous fluid drained for several months attributed to disruption of skin lymphatics. She continued compression and advanced wound care management and had complete healing and cessation of drainage. She experienced some loss of ankle flexion bilaterally due to splinting during healing and this partially improved with physical therapy. She was able to walk well unassisted, and while healing was challenging, she preferred the outcome of surgery to the experience of years with skin ulcers and is more functional than anticipated with the alternative outcome of bilateral amputation.

**Fig 2:**
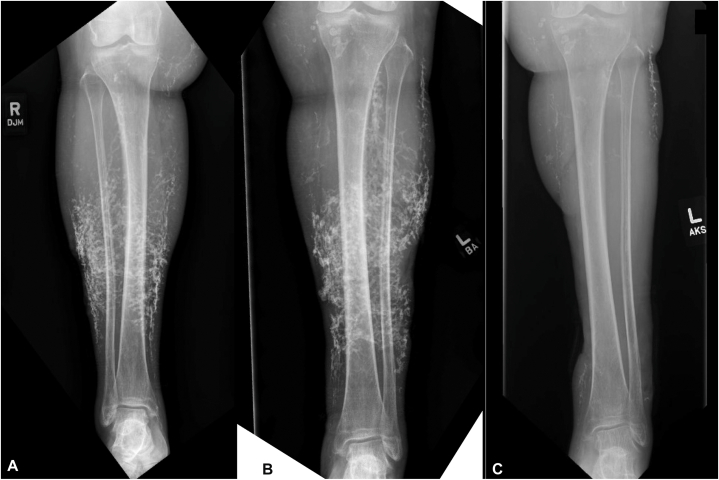
Radiographs of the right and left lower leg before surgery **(A** and **B)** and 17 months after surgery showing no calcinosis in the grafted areas on the left **(C)**. Post-operative right-leg radiographs were not obtained.

Approximately 1 year post surgery, the patient was transitioned to monthly intravenous immunoglobulin (IVIG) as an immunomodulatory, steroid-sparing strategy. Zoledronic acid was administered annually for 3 years (2 years prior to surgery and 1 year postoperatively), primarily for osteoporosis management rather than treatment of calcinosis cutis. Calcium-channel blocker therapy was continued without interruption. Nearly 2 years after surgery, she had not developed recurrent calcinosis or ulceration in the grafted areas (Fig 2, *D*).

## Discussion

Surgical intervention of CC can be helpful for localized areas that are painful or ulcerated. Calcinosis cutis of the hands and feet are more common areas to treat surgically due to the pain in these body areas with usual daily activities.^3^^,^^4^ Notably, the small size of the calcium deposits in these areas makes complete excision possible. Rarely has large excisional debridement with STSG been used for widespread CC. In 2 prior reports of unilateral lower-extremity surgical debridement with STSG, both patients recovered fully without recurrence of calcification.^5^^,^^6^ In our patient with extensive bilateral CC, circumferential surgical excision and STSG resulted in complete wound healing and avoided the alternative outcome of bilateral amputation. Although promising, caution must be exercised when determining surgical candidacy, as up to 70% of patients with systemic sclerosis may experience recurrent ulceration complications following surgery.^6^

Durable surgical outcomes in calcinosis cutis may depend not only on complete excision of calcified tissue but also on control of the underlying inflammatory disease process. In this patient, postoperative immunomodulation with intravenous immunoglobulin (IVIG) may have contributed to the sustained absence of recurrence, consistent with prior literature describing a therapeutic role for IVIG in calcinosis associated with autoimmune connective-tissue disease.^7^ As mentioned earlier, a partial debridement with skin grafting was performed earlier in this patient’s clinical course and prior to IVIG. It is notable that while the ulceration continued to expand circumferentially over areas of residual calcinosis, no calcinosis developed under the 3 × 3 cm skin graft in the several years before the definitive surgical treatment and before IVIG was started. This case suggests that patients with refractory calcinosis cutis and adequately controlled underlying systemic inflammation may represent a subgroup in whom aggressive surgical management can achieve durable benefit. This case supports that, with careful planning, extensive excision with STSG can be a viable treatment for recalcitrant CC with ulceration in lcSSc.

## Conflicts of interest

None disclosed.
